# Charcoal Pencils to Replace the Actual Cautery

**Published:** 1867-10

**Authors:** 


### *Charcoal Pencils to replace the Actual Cautery.*

In the May (1866) number of the same Journal, p. 358, is a formula for *Charcoal pencils to replace the actual cautery* recommended by M. Bretonneau: "Light powdered charcoal, 20 parts; nitrate of potash, 1½ parts; gum tragacanth, 5 parts; water, 24 parts: make a pilular mass, to be divided into cylinders of the size of an ordinary pencil, and about ten centimetres (nearly four inches) in length."

